# Harmonic analysis and FPGA implementation of SHE controlled three phase CHB 11-level inverter in MV drives using deterministic and stochastic optimization techniques

**DOI:** 10.1186/2193-1801-2-370

**Published:** 2013-08-05

**Authors:** Joshi Manohar Vesapogu, Sujatha Peddakotla, Seetha Rama Anjaneyulu Kuppa

**Affiliations:** Faculty of Electrical Engineering, JNTUA, Anantapur, AP India; Department of Electrical Engineering, JNTUCEA, Anantapur, AP India

**Keywords:** C-GA Technique, CHB inverter, FPGA based Xilinx’s Spartan-3A DSP controller, MPSO technique, NR technique, Selective harmonic elimination technique

## Abstract

With the advancements in semiconductor technology, high power medium voltage (MV) Drives are extensively used in numerous industrial applications. Challenging technical requirements of MV Drives is to control multilevel inverter (MLI) with less Total harmonic distortion (%THD) which satisfies IEEE standard 519-1992 harmonic guidelines and less switching losses. Among all modulation control strategies for MLI, Selective harmonic elimination (SHE) technique is one of the traditionally preferred modulation control technique at fundamental switching frequency with better harmonic profile. On the other hand, the equations which are formed by SHE technique are highly non-linear in nature, may exist multiple, single or even no solution at particular modulation index (M_I_). However, in some MV Drive applications, it is required to operate over a range of M_I_. Providing analytical solutions for SHE equations during the whole range of M_I_ from 0 to 1, has been a challenging task for researchers. In this paper, an attempt is made to solve SHE equations by using deterministic and stochastic optimization methods and comparative harmonic analysis has been carried out. An effective algorithm which minimizes %THD with less computational effort among all optimization algorithms has been presented. To validate the effectiveness of proposed MPSO technique, an experiment is carried out on a low power proto type of three phase CHB 11- level Inverter using FPGA based Xilinx’s Spartan -3A DSP Controller. The experimental results proved that MPSO technique has successfully solved SHE equations over all range of M_I_ from 0 to 1, the %THD obtained over major range of M_I_ also satisfies IEEE 519-1992 harmonic guidelines too.

## Introduction

Advancements of semiconductor switching devices like GTOs, IGBTs and IGCTs lead the pace of high power converters and MV Drives since 1980s. The MV drives cover the voltage ratings from 2.3 kV to 13.8 kV and power ratings from 0.4 MW to 40 MW. Medium voltage drives are extensively used in industrial applications such as steel rolling mills in the metals industry (Okayama et al. 1996), petrochemical industry (Rossmann & Ellis 1998), traction applications in the transportation industry (Bernert 2000), cement industry (Menz & Opprecht 2002) because of advantages such as: high efficiency, high reliability, effective fault protection, high dynamic performance, regenerative braking capacity, four quadrant operation and significant savings on energy cost (Bin Wu 2006).

However, a single power semiconductor switch alone cannot be connected to medium voltage grids (2.3kV, 3.3kV, 4.16kV or 6.9 kV). For this reason, different Multilevel Inverter topologies have emerged as a solution for working with higher voltage levels (Lai & Peng 1996). At present, there are three commercial topologies of Multilevel Inverters: Neutral point clamped (NPC) topology (Nabae et al. 1981), Cascaded H-bridge (CHB) topology (Meynard & Foch 1992) and Flying capacitor (FC) topology (Rodriguez et al. 2002; (Siva Kumar et al. 2010).

Among all the topologies, CHB topology has drawn significant importance because of the following advantages: (i) achievement of higher power levels by using lesser rating of semiconductor devices, (ii) no clamping diodes are required as in NPC topology, (iii) voltage balancing capacitors are not needed as in FC topology, (iv) Modularity in construction, (v) less dv/dt stresses which results in less Electro Magnetic Interference (EMI), (vi) lesser common node voltages (Renge et al. 2006) and producing output voltage with less Harmonic distortion (Malinowski et al. 2010). With an increasing of number of DC voltage sources, number of levels of output voltages increases which reduces the %THD.

However, as the number of levels increases, the control complexity increases. Since three phase CHB 11-level inverter is chosen for analysis, sixty semi conductor switches have to be controlled which further increases the control complexity. However, this problem is effectively addressed in this paper by successfully implementing MPSO technique in real time too.

In high power MV Drive applications, high converter efficiency with least %THD (which obeys IEEE 519-1992 harmonic guidelines) is of prime importance. Since, the switching losses are in direct relation with the modulation strategy used for controlling of MLI, they have been an active subject of research. High switching modulation control methods like Carrier based PWM techniques (Joshi et al. 2013) or Space vector modulation techniques leads to high switching losses and side bands around carrier frequency appears as lower order harmonics producing high %THD in output voltage and current (Samir Kouro et al. 2009).

Instead, low switching frequency modulation technique or fundamental switching frequency technique like Selective Harmonic Elimination (SHE) has been traditionally one of the widely used modulation method for control of MLI (Patel & Hoft 1973). SHE technique has attractive features such as: maintaining the good power quality, less switching losses, direct control over output voltage harmonics and the ability to leave triplen harmonics uncontrolled to take the advantage of circuit topology in three phase system (Al-Othman et al. 2007).

The equations which are produced by SHE technique are highly non-linear transcendental in nature that contains trigonometric terms and exhibit multiple sets of solutions. Thus, providing analytical solution (i.e. finding optimum switching angles) for SHE equations during complete range of M_I_ from 0 to 1, has been a main challenge for researchers over decades.

Several researchers have proposed many optimization techniques to solve non-linear transcendental SHE equations. These optimization techniques include deterministic methods like NR method (Patel & Hoft 1973; 1974), Predicted initial values (Sun & Grotstollen 1992), WALSH functions (Liang et al. 1997), Elimination theory (Chiasson et al. 2004), Resultant theory (Chiasson et al. 2005), Function minimisation (Vassilios et al. 2008) and stochastic optimization like modified species based particle swarm optimization(MPSO) (Tarafdar et al. 2009) or continuous genetic algorithm(C-GA) (Reza et al. 2011).

The main objective of this paper is to present comparative harmonic analysis for deterministic optimization technique like NR method and stochastic method like continuous C-GA and MPSO which are applied to solve SHE equations of three phase CHB 11-level inverter during whole range of M_I_ from 0 to 1 and to validate the simulated results with experimental approach.

This paper has been organized as follows: SHE technique and problem formation section briefly explains the SHE technique and formulation of objective function and cost function. Optimization techniques such as deterministic method like NR method and stochastic methods like C-GA and MPSO are briefly discussed in optimization techniques for Solving SHE Equations section. Results obtained from deterministic and stochastic optimization techniques are presented in simulation and analysis section. Real time implementation and comparative analysis are presented in hardware implementation section. Finally, conclusion section summarizes the merits and limitations of various algorithms and future scope of the work.

## SHE technique and problem formation

The selective harmonic elimination method is also called fundamental switching frequency method based on the harmonic elimination theory proposed by Patel et al. (Patel & Hoft, 1973; 1974). Selective harmonic elimination (SHE) technique is one of the preferred modulation methods at fundamental switching frequency for control of high power inverters since early 1960s and developed into matured form in 1970s. Theoretically it provides highest power quality at fundamental switching frequency in comparison to other existing PWM methods like space vector modulation and carrier based PWM techniques (Holmes & Lipo 2003).

**Figure 1:**
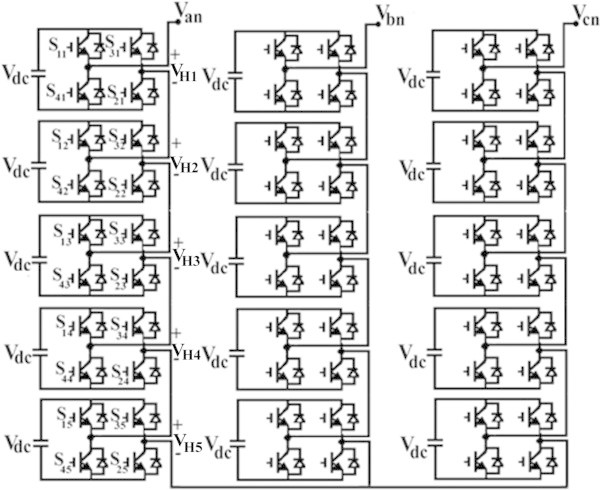
**Three phase cascade H-bridge 11-level inverter.**

**Figure 2:**
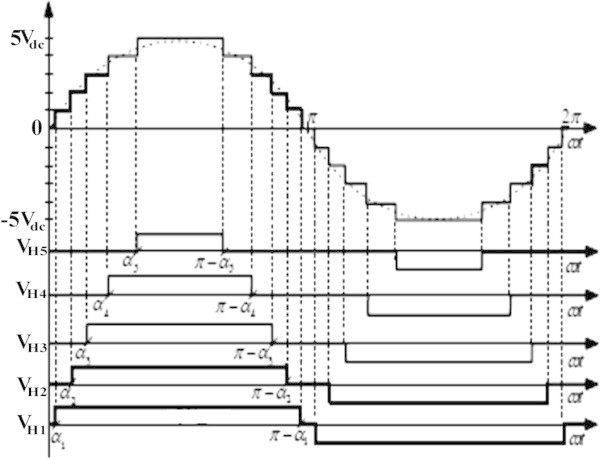
**Output phase voltage waveform of three phase CHB 11-level inverter.**

More over as the number of levels increases, the complexity of location of vectors become more complicated in space vector modulation whereas, in carrier based methods because of high frequency switching modulation converter efficiency decreases due to high thermal losses (Al-Othman et al. 2007). SHE method can also be called as programmed PWM method and has been widely used in applications such as power quality improvement techniques, MV Drives, High Voltage DC transmission (HVDC) systems and distribution systems (Cetin & Ermis 2009; Hammond, 1997). Figure 1 represents circuit configurations of three phase cascaded H-bridge inverter and Figure 2 represents per phase output voltage waveform of CHB 11-level inverter and its switching pattern.

Applying Fourier series for the stair case output voltage waveform of CHB 11-level inverter, as shown in Figure 1, it is given by1

where ‘s’ is the number of H-bridges connected in cascade per phase. Here, five single phase H-bridges are connected per phase and cascaded with each other. V_dc_ is input voltage of each H- bridge cell and total output voltage in each phase is given by2

In SHE technique, all the switching angles are limited in between 0° and 90° (0 ≤ α ≤ 90°). Because of odd quarter-wave symmetry of output voltage waveform, all even order harmonics are zero. Here, it is not necessary to eliminate triplen harmonics because they will be eliminated in the line to line output voltage.

Subsequently, from equation (1) the fundamental output voltage (V_1_) (when k = 1) in terms of switching angles is obtained by3

M_I_ is defined as the ratio of fundamental output voltage (V_1_) to maximum obtainable fundamental voltage V_1max_.

V_1max_ can be obtained by keeping all switching angles to zero degrees.4

M_1_ can be expressed as5

Since there are five H-bridges per phase for CHB 11-level inverter, five degrees of freedom are present. Among five degrees of freedom, one degree of freedom is used to produce fundamental voltage and remaining four degrees are used to minimize the lower order harmonics like 5^th^, 7^th^, 11^th^ and 13^th^. The main objective is to obtain maximum fundamental voltage by minimizing lower order harmonics as they significantly contribute to %THD. Combining the conditions in equations (1) and (5), SHE equations can be written as6

The main objective is to minimize the SHE equation set (6), which is expressed as7

Subject to8

### Cost/Fitness function

The main objective is to find a set of switching angles such that the magnitude of fundamental harmonic reaches a desired value, i.e V_1_^*^ by minimizing lower order of harmonics (i.e 5^th^, 7th, 11th and 13^th^). The fitness value is a measure of the appropriateness of a solution with respect to the original objective and the amount of infeasibility. The fitness function is formed by adding original objective function or fundamental component function to penalty function (Homaifar, Gi & Lai 1994; Michalewicz & Schoenauer, 1996).

For each solution (or each chromosome), the fitness function is calculated as follows910

Where, V_1_^*^ is the desired fundamental harmonic, ‘s’ is the number of switching angles and h_n_ is the order of n^th^ viable harmonic at the output of a three phase multi-level inverter e.g h_2_ = 5 and h_5_; = 13. Detailed explanation about functioning of fitness function is presented in Reza et al. 2011.

## Optimization techniques for solving SHE equations

In many industrial applications, it is required to operate the inverter over a range of M_I_ from 0 to 1. As it is mentioned, finding analytical (feasible) solution for SHE equations which gives least %THD has been a challenging task for researchers. Among the existing optimization techniques to solve non linear transcendental equations, deterministic methods like NR, stochastic methods like C-GA and MPSO are considered and comparative analysis have been carried out. This section briefly explains different optimization algorithms to solve SHE equations.

### Deterministic method

Newton-Raphson method is deterministic optimization technique which is extremely powerful and fast convergence iterative method to solve non linear transcendental equations (Patel & Hoft 1973). Newton's method was first described by Isaac Newton in 1969 and Joseph Raphson after twenty years later got close to Newtons approach but only for polynomials of degree 3, 4, 5 ……10. Finally in 1740, Thompson Simpson explained NR method as an iterative method to solve optimization problems by setting the gradient to zero (Peng et al. 1997).

Main steps for NR method are

- Step 1: Assume any random initial guess for switching angles (say α_0_).
- Step 2: Set M_I_ =0.
- Step 3: Calculation of F(α_0_),B(M_I_) and jacobian matrix J(α_0_).
- Step 4: Computation of error Δα.
- Step 5: Updating of the switching angles and Perform the transformation to bring switching angles in feasible range i.e. between zero and π/2.
- Step 6: Repeat the steps (3) to (6) for sufficient number of iterations to attain error goal.
- Step 7: Substitute α_0_ = α (k + 1).
- Step 8: Repeat steps (2) to (8) for whole range of M_I_.
- Step 9: Increment by a fixed step.
- Step 10: Repeat steps (2) to (10) for complete range of M_I_.

The proposed algorithm is implemented in MATLAB programming environment and complete analysis of functioning of algorithm is present in simulation and results section.

### Continuous genetic algorithms

In order to ease the complexity of controlling modern industries with multiple objectives and constraints, many researchers have developed biologically inspired algorithms and proved their effectiveness of control compared to derivative approaches. Genetic algorithm is one of the Stochastic optimization (SO) methods to solve non linear transcendental equations effectively. Stochastic optimization (SO) methods are optimization methods that generate and use random variables. GAs are a subclass of Evolutionary computing and are random search algorithms. Though, all minimum seeking algorithms uses the same basic approach of heading downhill from an arbitrary starting point but they differ in deciding in which direction to move and how far to move (Davis 1991).

After Successive improvements like increasing the speed of search process with good intelligence, without trapping at local minimum, powerful and widely accepted biologically inspired algorithm of Genetic Algorithm has been proposed by John Hallond in 1975 and finally it has been popularized by one of his student Goldberg who has solved the complex problem of control of gas-pipe line transmission for his dissertation work (Chamber 1995). Later it has been successfully implemented for solving number of engineering optimization problems due to the advantages such as Optimization with continuous or discrete variables, no need of calculus information, capability of dealing with a large number of variables, well suited for parallel computers, ability to find optimum global minimum instead of local minimum even in most complex objective functions (Sivanandam & Deepa 2011); (Deb 2001).

In this work, Continuous Genetic Algorithm has been used to solve non linear transcendental SHE equations during whole range of M_I_ from 0 to 1.

Steps involved in Continuous GA are as follows

- Step 1: Defining of optimization variables, cost function, cost.(cost minimization).
- Step 2: Generation of initial population.
- Step 3: Fitness/Cost evaluation.
- Step 4: Selection of Mates.
- Step 5: Mating.
- Step 6: Mutation.
- Step 7: Convergence check.
- Step 8: Repeat step (2) to step (7) until requirements met.

Detailed explanation of algorithm is presented in (Ozpineci et al. 2005).

### Modified particle swarm optimization

Swarm intelligence is defined as “. . . any attempt to design algorithms or distributed problem-solving techniques inspired by the collective behavior of social insect colonies and other animal societies.” (Karaboga & Akay 2009). One of the popular swarm-intelligence-based algorithm is the Particle Swarm Optimization (PSO) algorithm which was introduced by Eberhart and Kennedy in (1995,2002) (Kennedy & Eberhart, 1995); (2002). PSO is also a population-based stochastic optimization technique and is well adapted to solve complex optimization problems because of the advantages such as: with less computational effort, simplicity in computer coding, search techniques do not use gradient information but the values of objective function, initial guess is not needed like traditional iterative methods. Successful applications of PSO technique to engineering optimization problems are found in (Chan & Tiwari, 2007; Mohamed (Azab, 2009;); Hereford, & Siebold,); Hereford, & Siebold, ); Hereford, & Siebold,^2008^). Recently, Modified species –based PSO (MPSO) has proposed in (Tarafdar et al. 2009), which has better performance like fast convergence, less computational effort and calculation time than basic PSO. Hence, this algorithm has been considered for solving SHE equations set (6).

Main steps in algorithm are as follows.

- Step 1: Initialize population.
- Step 2: Repeat.
- Step 2: Calculate fitness values of particles.
- Step 3: Sort particles from the best-fitness value to least- fitness value.
- Step 4: Select species seed.
- Step 5: Assign each species seed identified as the *l*_best_ to all individuals identified in the same species.
- Step 6: Replace redundant particles in species.
- Step 7: Update particle positions according to (6) and (8).
- Step 8: Until requirements met.

The mathematical model which is represented in equation (11) indicates how particle updates itself in each iteration.11

V_i_^k^ : velocity of i^th^ particle at k^th^ iteration,

χ : Constriction co-efficient

c_1_, c_2_: The cogitative and social parameters

rand_1_, rand_2_ : random numbers between 0 and 1

x_i_^k^ : Current position of i^th^ particle at k^th^ iteration

p_besti_ : p_best_ of i^th^ particle

g_best_ : g_best_ of the group

Clerc & Kennedy proposed constriction co-efficient (χ) to guarantee the convergence of PSO algorithm.

Where *χ* is12

New position is calculated as the sum of the previous Position and the new velocity by (13)13

The complete explanation of MPSO algorithm is explained by Tarafdar et al. 2009.

## Simulation and results

In MATLAB programming environment, codes are developed for the proposed algorithms like NR method, C-Genetic Algorithm and Modified Particle Swarm Optimization to find near global optimum solutions for SHE equations and SIMULINK model is also developed to observe %THD from FFT analysis and results are discussed.

### Newton raphson method

In general, the main disadvantage of NR method is requirement of good initial guess which requires the past knowledge about convergence and good initial guess is not possible always. Hence, in this paper that drawback is eliminated by developing the algorithm which can work with any random initial guess. For an eleven level inverter, it is required to compute five switching angles at each M_I_ from 0 to 1 with an increment of 0.001.

**Figure 3:**
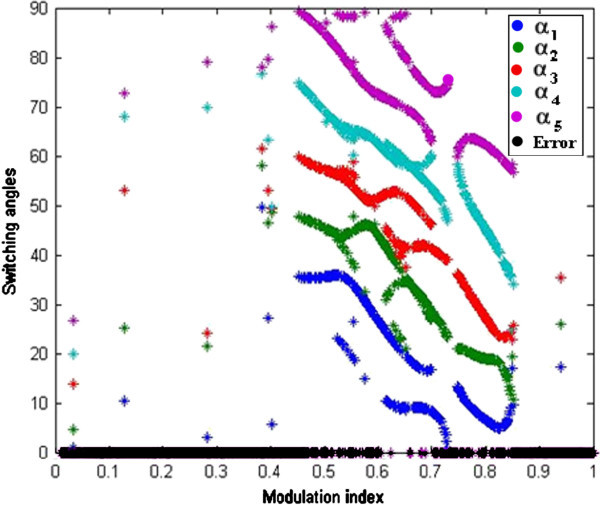
**Switching angles versus modulation index.**

**Figure 4:**
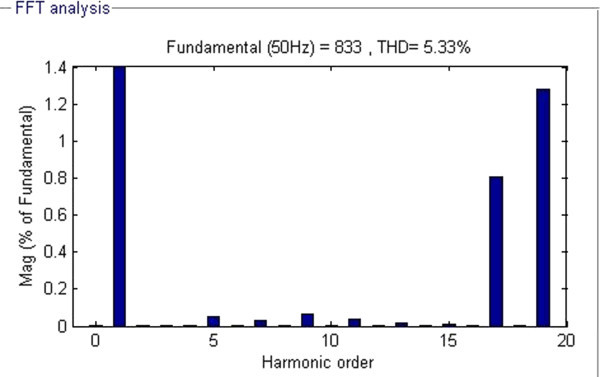
**FFT analysis representing order of voltage harmonics at M**_**I**_ **= 0.755.**

Random initial guess for five switching angles are in between 0° to 90°. Among five switching angles which are computed, one is required to produce fundamental output voltage and other four is used to eliminate lower order harmonics like 5^th^, 7^th^, 11^th^ and 13^th^. The simulation results obtained by implementing above algorithm are presented in Figures 3 and 4 and Table 1 respectively.

**Table 1:** **%THD** & **switching angles in degrees at various M**_**I**_

S. no	M_I_	α_1_	α_2_	α_3_	α_4_	α_5_	%THD
1.	0.460	35.46	47.39	59.32	73.95	88.73	8.81
2.	0.473	35.33	46.87	58.47	72.40	87.68	8.62
3	0.485	35.33	46.32	57.82	70.96	86.56	8.98
4	0.495	35.44	45.78	57.39	69.77	85.49	8.88
5	0.500	35.52	45.49	57.20	69.20	84.92	8.77
6	0.555	33.75	44.94	53.54	65.22	77.14	7.60
7	0.600	26.64	43.93	51.53	62.39	72.50	7.24
8	0.655	18.98	34.82	51.33	57.94	69.32	6.32
9	0.750	12.79	21.01	35.81	56.59	61.31	5.63
**10**	**0.755**	**11.66**	**20.93**	**34.83**	**54.41**	**62.67**	**5.33**
11	0.800	6.569	18.94	27.18	45.13	62.24	5.55

By observing the computed results from Figure 3, the feasible solutions (α_1_ α_2_, α_3_ α_4_ and α_5_) at various modulation indices can be broadly classified into three regions: 1) No solution region 2) Single solution region 3) Multiple solution region. In Figure 3, switching angles α_1_ α_2_, α_3_ α_4_ , α_5_ and error are represented in blue, green, red, green, violet and black colors respectively and all the switching angles are measured in degrees and represents the possible solution. It can be seen from Figure 3 that solutions does not exist at lower ends of M_I_ up to 0.460 and upper ends of the M_I_ after 0.85, as the error is not zero. Multiple solution sets exists for M_I_ = [0.510 0.569], [0.615 0.700]. Some solutions exist in very narrow range of M_I_ i.e. at [0.365], [0.387], [0.900], [0.933] and [0.982].

From Table 1, it is observed that the developed algorithm successfully solved the SHE equations during major range of M_I_ and interesting thing is that %THD produced during entire range of M_I_ are less than 8.98% and minimum %THD of value 5.33% is observed at M_I_ of value 0.755. From FFT analysis in Figure 4, it is clearly observed that all the desired lower order harmonics such as 5^th^, 7th, 11th and 13^th^ are minimized to a greater extent and the %THD value also satisfies IEEE 519-1992 harmonic guidelines too.

### Continuous genetic algorithm optimization technique

For all stochastic optimization technique computations in this paper, the values of the common parameters such as population size, maximum iteration number was considered same and the values are 300 and 1000 respectively.

**Figure 5:**
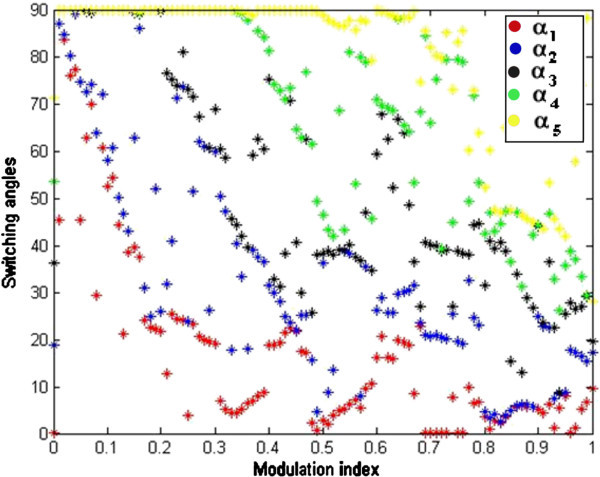
**Switching angles versus modulation index.**

**Figure 6:**
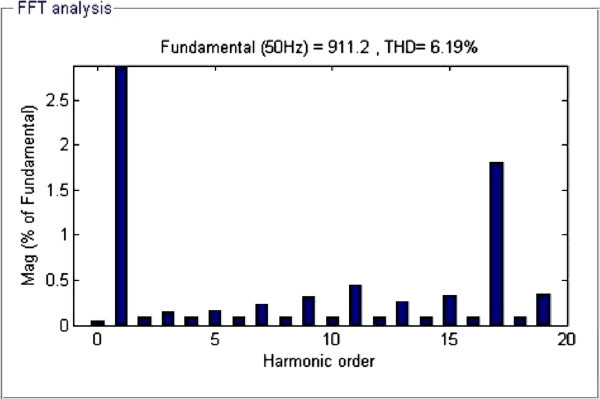
**FFT analysis representing order of harmonics at M**_**I**_ **= 0.97.**

Algorithm specific parameters which are considered for GA coding are as follows: Continuous / Real coded GA is employed for developing algorithm. Mutation rate of value 0.2, selection (fraction of population kept) value of 0.5 are considered. MATLAB programming environment is used to develop the algorithm and computed results are presented in Figures 5 and 6 and in Table 2 respectively.

**Table 2:** **%THD** & **firing angles in degrees at various M**_**I**_

S. no	M_I_	α_1_	α_2_	α_3_	α_4_	α_5_	%THD
1.	0.1	38.17	57.89	89.99	89.99	90	19.67
2.	0.2	10.57	21.68	89.77	89.84	89.84	12.81%
3.	0.3	19.16	38.61	59.91	89.98	89.99	11.33%
4.	0.4	6.840	18.79	31.41	75.18	89.99	11.93%
5.	0.5	4.671	30.83	37.44	48.55	89.42	11.12%
6.	0.6	11.64	33.58	33.74	56.79	78.11	10.67%
7.	0.7	0.001	11.45	20.78	40.12	82.00	9.17%
8.	0.8	6.62	23.10	44.42	54.37	63.57	8.59%
9.	0.9	4.85	5.243	24.95	34.12	44.23	7.30%
**10.**	**0.97**	**4.82**	**12.69**	**23.32**	**24.46**	**37.27**	**6.19%**
11.	1	3.24	9.496	17.17	28.03	28.14	6.85%

In order to overcome the problem of local trapping, the developed code is run for 10 times at each value of M_I_ and near global optimum solution is considered. From Figure 5, it is seen that, the developed C-GA optimization technique has successfully solved the SHE equations set for entire range of M_I_ from 0 to 1, which is unable to find in NR technique. The switching angles α_1_ α_2_, α_3_ α_4_ and α_5_ are in degrees and represented in red, blue, black, green and yellow colors respectively and represents possible solutions obtained from C-GA technique.

Table 2 presents feasible solutions (switching angles) at various M_I_ from 0 to 1. As the M_I_ increases %THD decreases and minimum %THD of value 6.19% has occurred at M_I_ of 0.97. FFT analysis at M_I_ of value 0.97 is presented in Figure 6. It reveals that the targeted lower order harmonics of 5^th^, 7^th^, 11^th^ and 13^th^ are significantly minimized and the value of %THD is 6.19%, which also complies with IEEE 519-1992 harmonic guidelines. M_I_ at which maximum fundamental voltage is achieved, %THD is found to be significantly minimum of value 6.85%. Thus, the simulation results validate the effectiveness of C-GA optimization technique.

### Modified particle swarm optimization technique

MATLAB code is developed using MPSO technique by considering algorithmic specific parameters as follows: cognitive parameter (c_1_), social parameter (c_2_) are considered as 1 and 3.5 respectively. *r*1 and *r*2 are random values uniformly distributed within [0, 1].

**Figure 7:**
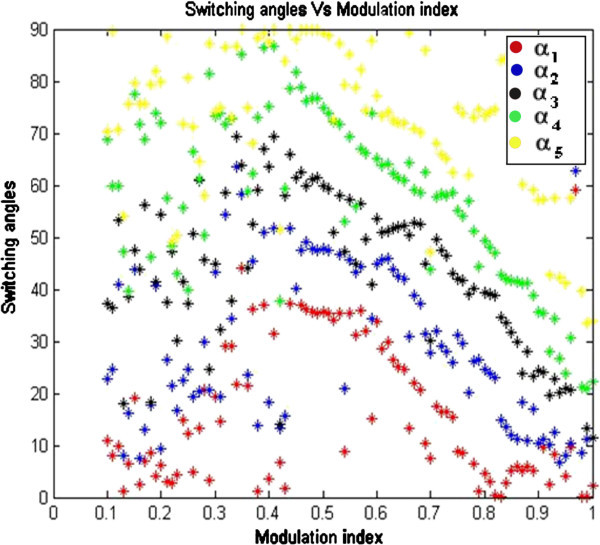
**Switching angles versus modulation index.**

**Figure 8:**
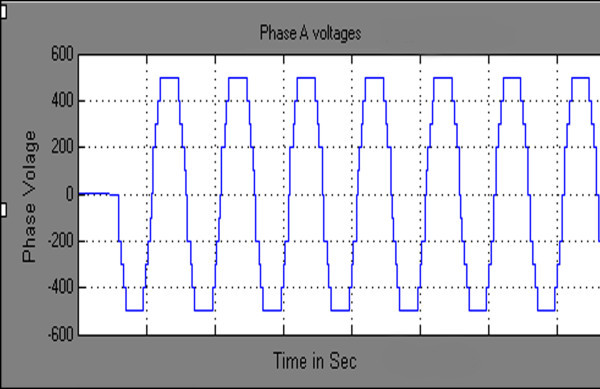
**Output phase voltage waveform of three phase CHB 11-level inverter.**

**Figure 9:**
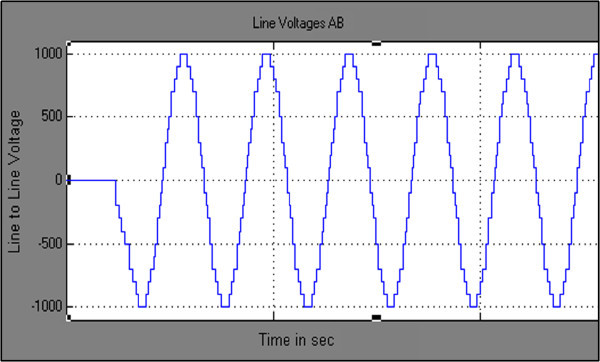
**Output line to line voltage waveform of three phase CHB 11-level inverter.**

**Figure 10:**
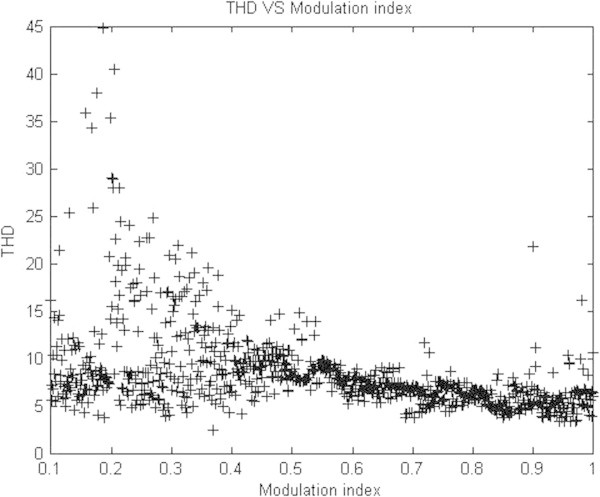
**THD Vs modulation index.**

**Figure 11:**
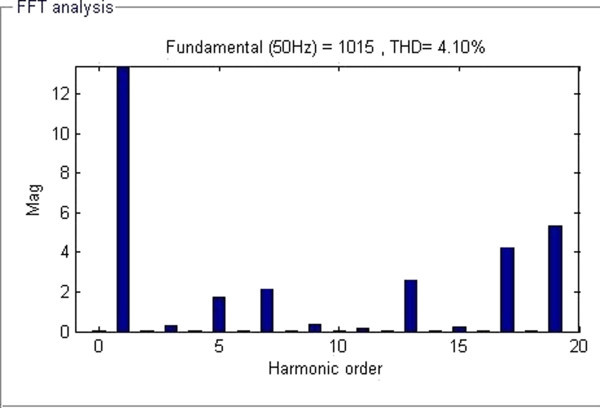
**FFT analysis representing order of voltage harmonics at M**_**I**_ **= 1.0.**

Maximum velocity of swarm particles (*v*_*max*_*),* number of spices at start, number of spices at last and the constant parameter *vrs* which indicates the variation of *r*_*s*_ (nichie radius) at each iteration step are considered as 0.2, 50, 0 and 0.02 respectively as recommended in (Tarafdar & Taghizadeh, 2009). The developed algorithm is run in MATLAB software at each M_I_, the obtained feasible switching angles are given to SIMULINK model of chosen configuration to compute %THD. Computed results are represented in Figures 7, 8, 9, 10 and 11 and in Table 3.

**Table 3:** **%THD** & **switching angles in degrees at various M**_**I**_

S. no	M_I_	α_1_	α _2_	α _3_	α _4_	α _5_	%THD
1.	0.2	58.91	74.73	78.68	82.30	89.98	21.29
2.	0.3	55.91	63.45	65.49	78.21	89.99	14.36
3.	0.4	45.23	51.18	67.29	75.62	88.20	10.67
4.	0.5	35.55	45.36	57.12	69.97	84.68	8.60
5.	0.6	10.79	29.88	46.18	63.11	87.56	8.14
6.	0.7	16.63	26.60	45.84	60.13	62.73	6.44
7.	0.75	11.56	20.93	34.77	54.26	62.75	4.98
8.	0.8	6.884	19.01	27.80	45.77	62.52	4.69
9.	0.9	2.68	10.97	16.59	26.69	39.69	4.60
**10.**	**1.0**	**6.129**	**8.00**	**12.68**	**21.67**	**29.30**	**4.10**

From Figure 7, it is clearly observed that, the developed MPSO algorithm has successfully solved SHE equations set (6) during entire range of M_I_. The switching angles α_1_ α_2_, α_3_ α_4_ and α_5_ are in degrees and represented in red, blue, black, green and yellow colors respectively and represents possible solutions obtained from MPSO technique. It is seen that feasible solutions obtained are multiple at some values of M_I_ and single at some values of M_I._ In the case of multiple solutions, the feasible solutions which produce least %THD are considered.

Figures 8 & 9 represents simulated output phase voltage which contains eleven steps and line to line voltage wave forms of three phase CHB 11-level inverter. Table 3 shows, feasible switching angles and %THD at each value of M_I_. As the value of M_I_ has increased linearly from 0 to 1 in steps of 0.1 the %THD decreases. Figure 10 represents the graph of %THD at various M_I_. It can be seen that the value of %THD over a major range of M_I_ (greater than 0.5) found to be nearer to 6.0% and also satisfies IEEE 519-1992 harmonic guidelines.

From Table 3, the minimum value of %THD of 4.10% is obtained at M_I_ of value 1.0 and FFT analysis at that M_I_ is also shown in Figure 11. It can be seen that all the lower order of harmonics such as 5^th^, 7th, 11^th^ and 13^th^ are significantly reduced to a greater extent and the value of %THD is 4.10%. Thus, it proves the effectiveness of proposed MPSO algorithm for solving this particular optimization problem.

## Hardware implementation

**Figure 12:**
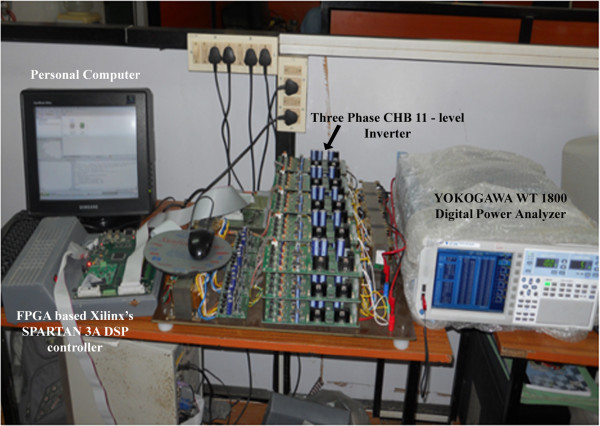
**Experimental set up of three phase CHB 11-level inverter to validate MPSO.**

To validate the effectiveness of proposed MPSO algorithm, a low power prototype of Three phase CHB 11-level inverter is considered and complete experimental set up arrangement is as shown in Figure 12. Hardware implementation consists of five single phase H-bridge inverters with equal independent DC sources of value 22V and they are connected in cascade with each other. In total, this set up consists of fifteen independent DC sources and sixty- IRF 840, 8A, 500V, 0.850 Ohm, Power MOSFETs. FPGA based Xilinx’s Spartan-3A DSP controller is used to interface MATLAB programming part and to generate gating signal to sixty MOSFETs. YOKOGAWA WT 1800 digital power analyzer is used to observe FFT analysis and output voltage waveforms. The digital gate firing pulse generation for sixty MOSFETs are implemented on Xilinx’s Spartan-3A DSP controller and it is low-cost evaluation platform for Spartan-3A DSP FPGA designs. Since, available microcontroller or DSP solutions have insufficient PWM hardware peripheral resources to enable sophisticated control of the chosen inverter, an Field Programmable gate array (FPGA) was selected for experimenting on the SHEPWM control technique because of the large number of PWM channels required for control of three phase CHB 11-level inverter. The internal architecture of the FPGA is dynamically configured to route signals to the internal logic gate resources to match the desired digital circuit design. The FPGA design for the experimental inverter has been written in VHDL. This is programmed via a personal computer and configures the FPGA once power is cycled on the development board.

**Figure 13:**
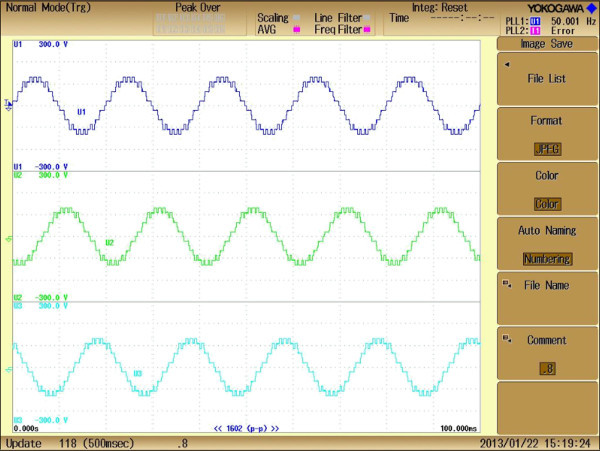
**Experimental output phase voltage waveforms of three phase CHB 11-level inverter.**

**Figure 14:**
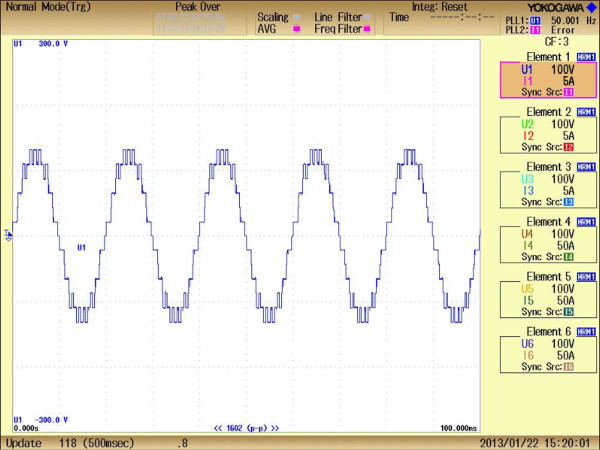
**Experimental line to line output voltage waveform of three phase CHB 11-level inverter.**

**Figure 15:**
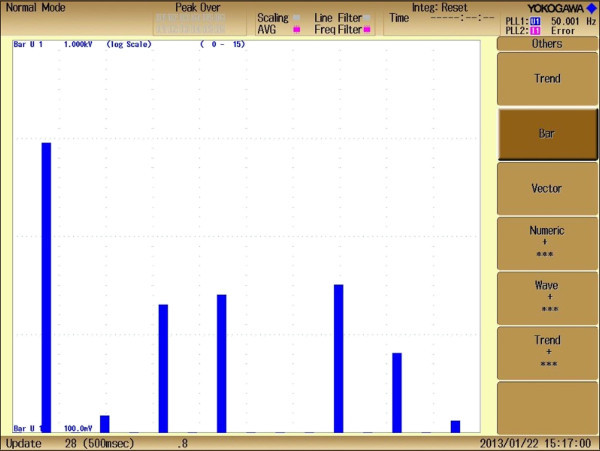
**Experimental FFT analysis (bar) representing order of the harmonics.**

**Figure 16:**
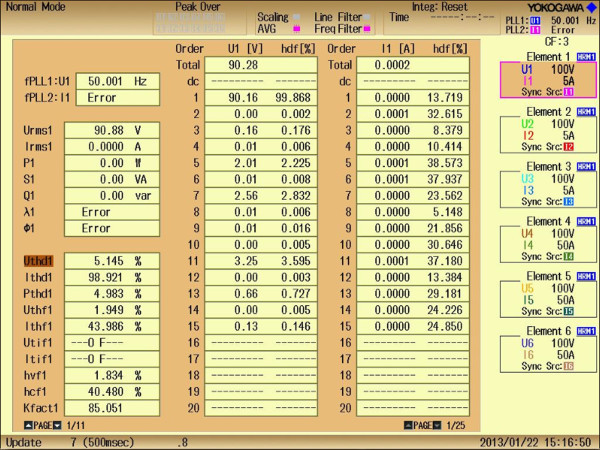
**Experimental FFT analysis list representing %THD value as 5.145%.**

The developed MPSO algorithm is run at various M_I_ from 0 to 1.0 and computed results are presented in Figures 13, 14, 15, 16 and Table 4. The comparative analysis of %THD at various M_I_ from 0 to 1.0 of simulation and experimental approach are listed in Table 4. Figures 13 and 14 represents the experimental output phase which contains eleven steps and line to line voltages of chosen inverter.

**Table 4:** **%THD values of simulation and experiment approach at various M**_**I**_

S. no	M_I_	% THD	% THD
		(Simulation)	(Experimental)
1.	0.2	21.29	22.80
2.	0.3	14.36	15.64
3.	0.4	10.67	11.16
4.	0.5	8.60	10.48
5.	0.6	8.14	10.10
6.	0.7	6.44	7.46
7.	0.75	4.98	6.80
8.	0.8	4.69	5.92
9.	0.9	4.60	5.17
**10.**	**1.0**	**4.10**	**5.14**

From experimental results, it is clearly observed that %THDs obtained at various M_I_ validates the simulation results and are very close with each other. It is also observed for the M_I_ of value greater than 0.5, %THD values are less and significant less values of %THDs are observed at 0.7, 0.75, 0.8, 0.9 and 1.0. As the value of M_I_ increases linearly from 0 to 1.0, the %THD value decreases and minimum of value 5.145% has occurred at M_I_ of 1.0

Figure 15 represents experimental FFT analysis representing order of harmonics at M_I_ of 1.0. It can be seen that, desired lower order harmonics such as 5^th^, 7^th^, 11^th^ and 13^th^ are significantly minimized and the value of %THD at that M_I_ is found to be 5.145%, which can be seen clearly from experimental FFT analysis list as shown in Figure 16. This value of %THD is significantly minimum and complies with IEEE 519-1992 harmonic guidelines. In addition to that the values of %THD which are obtained above M_I_ of value 0.7 comply with IEEE 519-1992 harmonic guidelines too.

## Conclusions

This paper mainly focused on developing efficient, rugged algorithm with less computational effort to solve SHE equations set during entire range of M_I_ from 0 to 1.0 to get feasible switching angles, in order to obtain less %THD which satisfies IEEE 519-1992 harmonic guidelines. A three phase CHB 11-level inverter has been chosen for analysis. In order to solve SHE optimization problem, deterministic method like NR method, stochastic optimization methods like C-GA and MPSO techniques have been applied.

By observing the computational results from NR method, though the drawback of good initial guess has been overcome by any random initial guess here, NR algorithm could not solve SHE equations set during lower ends of M_I_ up to 0.460 and upper ends of M_I_ greater than 0.850. But the algorithm is rugged and has effectively solved SHE equations from M_I_ of value 0.460 to 0.850 with significantly less %THD. The maximum value of %THD obtained is 8.9% and minimum is 5.33%. Also, in NR method by comparing FFT analysis obtained from all the techniques, all the lower order harmonics like 5^th^, 7^th^, 11^th^ and 13^th^ are completely minimized which could not be observed in optimization techniques mentioned here.

C-GA has effectively solved SHE equations set during complete range of M_I_ from 0 to 1, which could not be solved by NR technique. The values of %THDs are significantly minimum has observed at above 0.7 M_I_ of value and least value of 6.10% has occurred at of value 0.97 M_I_. The magnitude of fundamental voltage at which minimum %THD has occurred is much greater than that obtained in NR technique. Though, it could overcome the drawbacks of NR methods, the complexity of writing long coding is more because of processes such as cross over, mutation etc. It is observed for this problem, in order to eliminate the problem of local trapping, the algorithm needs several runs, say 10 and minimum %THD among them has to be considered.

Proposed stochastic technique like MPSO algorithm has successfully solved SHE equations set during complete range of M_I_ from 0 to 1. Implementation of MPSO algorithm is easy as the algorithm is robust and it effectively provides near global optimum solutions. The values of %THD above 0.4 Modulation indices are minimum and significantly less %THDs are observed above 0.7 M_I_. The least %THD of value 4.10% is obtained at M_I_ of 1.0. Thus, the magnitude of fundamental voltage at which least %THD has occurred is more compared to above mentioned techniques. Hence, this proposed MPSO technique has been considered for experimental validation.

FPGA Xilinx’s SPARTAN 3-A DSP controller is used to generate the required sixty gate pulses for chosen three phase CHB 11-level inverter. From experimental results listed in Table 4, it is clearly observed that %THDs obtained at different M_I_ from 0 to 1.0 are very close with each other and the %THDs obtained above M_I_ of value 0.7, comply with IEEE 519-1992 harmonic guidelines too. Least %THD of value 5.145% is obtained at M_I_ of value 1.0 from experimental analysis and from simulation approach is 4.10%. Since the value of %THD obtained from experimental and simulation analyses are nearly equal, thus it proves the effectiveness of proposed algorithm in real time too.

From above analysis, it is understood that, for this optimization problem among all the mentioned optimization techniques, MPSO has successfully solved SHE equations and the experimental results also validates the effectiveness of proposed MPSO algorithm and %THDs obtained over a range of M_I_ also comply with IEEE 519-1992 harmonic guidelines too. Hence, this can be successfully used in applications such as MV Drives, renewable sources, hybrid electrical vehicles and power quality improvement devices.

On the other hand, in all stochastic optimization techniques, algorithm specific parameters such as mutation rate, social parameter, cognitive parameter and constriction factor are needed. However, during recent advancements in optimization techniques, teaching learning based optimization (TLBO) algorithm has newly introduced (Rao, & Patel 2012). TLBO algorithm does not use such specific algorithm parameters. Thus, it may be implemented for solving SHE optimization problem which may reduce the burden further in developing code.

## Authors’ information

JM received B.Tech degree in Electrical & Electronics Engineering from Nagarjuna University, Guntur, AP, India in 2000 and M.Tech degree in Power Electronics from Visveswaraiah Technological University, Belgaum, KA, India in 2004. Presently he is pursuing Ph.D degree from J.N.T.U. College of Engineering, Anantapur AP, India. Currently he is with the Dept of Electrical and Electronics Engineering, Guntur Engineering College, Guntur,AP,India. His area of research includes multilevel inverter control, Industrial Drives and Optimisation Techniques. He is Life member of ISTE.

SP is presently working as Professor & HOD of Electrical and Electronics Engineering department, JNTUCEA, Anantapur, AP, India. She completed her B.Tech, M.Tech and Ph.D. degrees in Electrical Engineering from J.N.T.U.Anantapur, Anantapur, AP, India, in 1993, 2003 and 2012 respectively. Her area of interest includes Reliability Engineering with emphasis to Power Systems and Real time Energy Management.

KSR has completed his B.Tech, M.Tech and Ph.D. degrees in Electrical Engineering from JNTUH, Hyderabad, AP, India in 1982, 1985 and 1999 respectively. Currently he is Principal JNTU College of Engg, Anantapur, India. His research interest includes Power Systems and Intelligent Techniques. He is Fellow of Institution of Engineers (I), Life Member of ISTE and Indian Society of Power Engineers (ISPE).
